# 

**Published:** 1781-02

**Authors:** 


### DEATHS.

*Nov.* 29, 1780, at Leyden, aged 76 years, Jerom David Gaubius, M. D. Honorary Profeſſor of Chemiſtry and the Practice of Phyſic, Firſt Phyſician to the Stadtholder, F. R. S. of London, and of many other ſimilar inſtitutions in different parts of Europe. He was a native of Heidelberg in Germany, and was the favourite pupil of Boerhaave, who reſigned the chemical chair in his favour in 1735, as he himſelf did in 1775 in favour of his nephew Dr. Hahn. His works are few in number, but his *Inſtitutiones*

*Pathologiæ Medicinales* alone are a ſufficient proof of his great abilities as a medical writer.

*Dec.*—At Vienna, aged 27 years, Maximilian Fellner, M. D. Profeſſor of Phyſiology in the Univerſity at that place.

6.—At Verſailles, aged 78, Joſeph Lieutaud, M. D. Counſellor of State, and Firſt Phyſician to the King of France, Member of the Royal Academy of Sciences at Paris, of the Royal Society of London, &c. He was a native of Aix in Provence, and in 1774 ſucceeded M. Senac as archiater. He acquired conſiderable reputation as a practical writer by his *Synopſis Univerſæ Praxeos Medicæ*, 2 vols. 8vo.; and as an anatomiſt, by his *Eſſais Anatomiques* (a late edition of which is much improved by M. Portal) and his *Hiſtoria Anatomico-Medica ſiſtens numeroſiſſima cadaverum humanorum extiſpicia.* 2 vols. 4to.

*Feb.*—1781.—At Cheſterfield in Derbyſhire, Mr. John Willot, ſurgeon and apothecary.

15.—At Darlington, in the county of Durham, John Trotter, M. D. author of an inaugural diſcourſe, "*de Morbo Venereo*," printed at Leyden in 1757.

SECTION

### SECTION IV.

### MONTHLY CATALOGUE.

1. MODERN improvements in the Practice of Surgery, by Henry Manning, M. D. 8vo. 423 pages, London; 1780.

There is no ſuch perſon as Henry Manning, M. D.—In our laſt monthly catalogue we announced another work under the ſame name, entitled, *Modern Improvements in the Practice of Phyſic.* The two volumes may be conſidered as uſeful compilations from the beſt modern Engliſh medical and ſurgical writers; but they would have been more valuable if the editor had drawn his materials from a greater variety of ſources, and given his readers abſtracts of foreign works of merit that are not ſufficiently known in this country. At the end of the medical volume, we meet with ſome account of the remedies that have been introduced into practice of late years; but what is ſaid on theſe ſubjects is in general not ſufficiently ſatisfactory; the account given of the columbo root, for inſtance, is comprized in half a dozen lines.

2. *C. E. Gellert,* Anfangſgründe zur Metallurgiſchen chymie in einem theoretiſchen und praktischen Theil. mitkupfern. Zweyte vermehrte und verbesserte Ausgabe. *i.e.* Elements of the Theory and Practice of Metallurgical Chemistry, by *C. E. Gellert*, with copper-plates, second edition, corrected, with additions, 8vo. Leipsic, 498 pages.

The first edition of this work was published in 1750. In the present edition the author, who is inspector of the mines in Saxony, has availed himself of all the improvements that have been made in metallurgical chemistry since that period. Mr. Gellert informs us, that he has sometimes dissolved copper filings, and likewise thin plates of copper, by mixing them with charcoal, and exposing them, in close vessels, to a long continued moderate heat. By means of this process he has obtained sometimes a grey, and sometimes a beautiful red *vitrum cupri*, which was increased $\frac{1}{8}$ part in its weight. In a very strong fire this substance was again deprived of its phlogiston and a pure malleable copper regenerated. From this circumstance it may be concluded that phlogiston alone is sometimes employed by nature to mineralize copper, and perhaps some other metals likewise. Our author does not consider the regulus obtained from cobalt as a peculiar semi-metal, but as a composition of iron and arsenic with another substance as yet unknown; and he is of opinion that the blue colour cobalt imparts to glaſs, depends at leaſt in a certain degree on iron.

3. Anfangſgründe zur Probierkunſt, *i. e.* Elements of the Art of aſſaying Metals, by *C. E. Gellert*, a new edition, with additions. 8vo. Leipſic. 176 pages, with copper-plates.

This work, as well as the one laſt mentioned, has received many valuable additions from the author's own experience.

4. *William Cullen*, D. A. D. &c. Anfangſgründe der Praktiſchen Arzneywiſſenſchaft, *i. e.* Firſt Lines of the Practice of Phyſic, by William Cullen, M. D. &c. Parts 1 and 2, 8vo. Leipſic.

5. *Samuel Foart Simmons*, D. A. D. &c. Anatomie des Menſchlichen Körpers, oder Beſchreibung des Baues deſſelben. Aus dem Engliſchen, und mit des überſetzen eigenen Anmerkungen vermehrt und verbeſſert, *i. e.* The Anatomy of the Human Body, or a Deſcription of its Structure, by *Samuel Foart Simmons*, M. D. &c. tranſlated from the Engliſh, with notes by the Tranſlator. 8vo. Leipſic.

6. Obſervations ſur la Rage; ſuivies de Reflexions critiques ſur les Preſervatiſs de cette Maladie, par *M. le Roux*, Maitre en Chirurgie, Aſſocié de l'Academie Royale des Sciences de Dijon, et Chirurgien

Chirurgien Major de l'Hopital General de la meme ville. Dijon, 8vo, 52 pages.

The author of this pamphlet (which, though it contains nothing new, is judicioufly written) is very properly of opinion that none of the remedies that have hitherto been recommended with a view to prevent the hydrophobia, are deferving our confidence. He thinks with fome other late writers that our principal attention ought to be directed to the wound through which the poifon may be expected to enter the habit, and accordingly recommends fcarification, topical bleeding, the cautery, &c. in order to prevent an abforption of the virus.

7. Obfervations rares de medecine, d'anatomie, et de chirurgie, traduites du Latin de Vander Wiel, par *M. Planque*, M. D. avec figures. Paris, 12mo. 2 Vols. containing each about 550 pages.

8. Experiences, et obfervations fur differentes efpeces d'air, ouvrage traduit de l'Anglois de *M. Prieftley*, Docteur en Droit, membre de la Société Royale de Londres. Par *M. Gibelin*, M. D. Membre de la Société Medicale de Londres. Tomes IV and V. Paris, 8vo.

9. Inftruccion curativa de los Tabardillos; par el *Don Jofef Amar*, M. D. &c. *i. e.* Remarks on on the cure of eruptive diseases, by *Don Joseph Amar*, M. D. Physician to his Majesty, Counsellor of the Royal Tribunal of Physic, first Physician for the kingdom of Navarre, Member of the Royal Society of Sciences at Seville, and Vice President of the Royal Medical Academy at Madrid. 4to, Madrid.

In this work, which consists of 327 pages, we read much of putridity and malignancy, of crudity and coction. Lewis de Toro, John de Carmona, and other Spanish medical writers are the most frequently quoted. The work closes with an account of the different epidemics that have prevailed in Spain.

10. Instruccion curativa y preservativa de dolores de costado y pulmonias; *i. e.* Remarks on the cure of diseases of the breast and lungs. *By the same.* 4to. Madrid, 204 pages.

The author divides pleurisy into the ascending and descending, and from these distinctions derives the indications for bleeding or purging in this disease.

11. Les grands remedes contre la rage, l'épilepsie, les vertiges et vapeurs qui ont atteints a ce mal, et autres infirmités; par M. *Le Jayant*, Curé de Nôtre Dame de la Quinte près de Mans. 8vo. Mans.

This

This Curate of La Quinte may not improperly be ranked as a medical writer with our country-man Mr. Wesley.

12. Histoire Medicale des Maladies dysenteriques qui affligerent la province de Maine en 1779; moyens convenables pour combattre avec succés le mal principal et les accidens qui en sont la suite; par M. *Vetillard*, M. D. Membre du College des Medecins de Mans. 12mo. Mans. 74 pages.

This seems to be the production of an experienced and judicious practitioner.

13. De China China in Synochis Putribus animadversiones *Petri Joannis Vastpania* Amplissimi Taurinensis Medicorum Collegii Socii, nec non Medici in Nosocomio urbis Majori Vicarii. Taurinii, 8vo, 152 pages.

The author of this Work condemns the Peruvian bark, but his arguments are by no means well founded.

14. Le Citoyen Dentiste, ou l'art de seconder la Nature pour se conserver les dents, et les entretenir propres. Ouvrage Moderne, a la portée de tout le Monde. Par *M. Hubert*, Chirurgien dentiste, recû au College Royal de Chirurgie de Paris, dentiste pensionné de la Ville. 12mo. Lyons, 95 pages.

